# Acceptability of lifelong treatment among HIV-positive pregnant and breastfeeding women (Option B+) in selected health facilities in Zimbabwe: a qualitative study

**DOI:** 10.1186/s12889-017-4611-2

**Published:** 2017-07-25

**Authors:** Addmore Chadambuka, Leila Katirayi, Auxilia Muchedzi, Esther Tumbare, Reuben Musarandega, Agnes I. Mahomva, Godfrey Woelk

**Affiliations:** 1 Elizabeth Glaser Pediatric AIDS Foundation, 107 King George Road, Avondale, Harare, Zimbabwe; 2 Elizabeth Glaser Pediatric AIDS Foundation, Washington DC, USA

**Keywords:** Acceptability, Option B+, Life-long treatment, Male partner support

## Abstract

**Background:**

Zimbabwe’s Ministry of Health and Child Care (MOHCC) adopted 2013 World Health Organization (WHO) prevention of mother-to-child HIV transmission (PMTCT) guidelines recommending initiation of HIV-positive pregnant and breastfeeding women (PPBW) on lifelong antiretroviral treatment (ART) irrespective of clinical stage (Option B+). Option B+ was officially launched in Zimbabwe in November 2013; however the acceptability of life-long ART and its potential uptake among women was not known.

**Methods:**

A qualitative study was conducted at selected sites in Harare (urban) and Zvimba (rural) to explore Option B+ acceptability; barriers, and facilitators to ART adherence and service uptake. In-depth interviews (IDIs), focus group discussions (FGDs) and key informant interviews (KIIs) were conducted with PPBW, healthcare providers, and community members. All interviews were audio-recorded, transcribed, and translated; data were coded and analyzed in MaxQDA v10.

**Results:**

Forty-three IDIs, 22 FGDs, and five KIIs were conducted. The majority of women accepted lifelong ART. There was however, a fear of commitment to taking lifelong medication because they were afraid of defaulting, especially after cessation of breastfeeding. There was confusion around dosage; and fear of side effects, not having enough food to take drugs, and the lack of opportunities to ask questions in counseling. Participants reported the need for strengthening community sensitization for Option B+. Facilitators included receiving a simplified pill regimen; ability to continue breastfeeding beyond 6 months like HIV-negative women; and partner, community and health worker support. Barriers included distance of health facility, non-disclosure of HIV status, poor male partner support and knowing someone who had negative experience on ART.

**Conclusions:**

This study found that Option B+ is generally accepted among PPBW as a means to strengthen their health and protect their babies. Consistent with previous literature, this study demonstrated the importance of male partner and community support in satisfactory adherence to ART and enhancing counseling techniques. Strengthening community sensitization and male knowledge is critical to encourage women to disclose their HIV status and ensure successful adherence to ART. Targeting and engaging partners of women will remain key determinants to women’s acceptance and adherence on ART under Option B+.

## Background

### HIV in Zimbabwe

Zimbabwe is one of the countries with the highest HIV burden in sub-Saharan Africa countries [1]. According to the 2015 Spectrum modelling national HIV estimates, HIV prevalence among adults 15–49 years in Zimbabwe in 2014 was 14.7% [2]. Among those most affected are pregnant women: with an estimated prevalence of 16.7% [2]. In 2014, an estimated 66,014 pregnant women were in need of antiretroviral therapy (ART) for prevention of mother to child transmission (PMTCT) of HIV [2], while 57,991 (88%) pregnant and lactating women were initiated on antiretroviral therapy (ART) in that year [2].

### PMTCT in Zimbabwe

#### Option A

Mother-to-child HIV transmission of HIV (MTCT) refers to transmission of HIV from an HIV-positive woman to her child during pregnancy, labor, delivery or breastfeeding. The cascade of HIV prevention of mother-to-child transmission (PMTCT) interventions includes antenatal care services, HIV testing during pregnancy, use of antiretroviral therapy (ART) by the woman during pregnancy and the mother and her newborn baby during the breastfeeding period, safe delivery practices, safe infant feeding practices, early HIV testing of the infant and other post-natal healthcare services [3]. In May 2010, Zimbabwe adopted the World Health Organization (WHO) 2010 guidelines (Option A). Under Option A, eligibility for antiretroviral prophylaxis or therapy was determined by CD4 count (immunologic condition) and WHO clinical staging. Women with a CD4 count ≤350 cells/mm^3^ or WHO stage 3/4 were initiated on lifelong ART. Women with a CD4 > 350 cells/mm^3^, and WHO stages 1 and or 2 were initiated on antiretroviral prophylaxis (zidovudine from 14 weeks gestation, with single-dose nevirapine [NVP] during labor and zidovudine and lamivudine given starting in labor through 7 days post-delivery; the breastfeeding infant receives infant NVP syrup from birth until 1 week after cessation of breastfeeding.

#### Option B+

The 2013 WHO consolidated guidelines recommended that all HIV-positive pregnant and breastfeeding women begin triple ART and continue ART until at least breastfeeding cessation (called Option B), with administration of lifelong treatment regardless of their CD4 count or clinical staging in high HIV burden settings (Option B+) [4]. Unlike Option A, which required the baby to be on NVP throughout the breastfeeding period, Option B/B+ only required the baby to be on NVP for 6 weeks after birth. Zimbabwe adopted the Option B+ guidelines in 2014.

Malawi, the first country to implement Option B+, saw a fivefold increase in the number of women initiating ART [5] and 80% retention rate of women who initiated Option B+ in antenatal care (ANC), which was an important reason for Zimbabwe’s choice of the Option B+ policy [6]. Other reasons for adopting Option B+ included Zimbabwe’s high fertility rate – with Option B, women would be frequently starting and stopping ART, creating risk of drug resistance [7]. Option B+ would provide protection against mother to child transmission of HIV in current and future pregnancies, reduction of transmission of HIV to discordant sexual partners, and increased access to early treatment for women in need of antiretroviral therapy for their own health [5].

At the time that Zimbabwe made a decision to adopt Option B+, there was scant literature exploring the acceptability of lifelong antiretroviral therapy for HIV-positive pregnant women who did not meet the eligibility criteria for treatment for their own health. Available literature explored the acceptability of ART to the general population. Anecdotal evidence from other countries suggested that acceptability of lifelong treatment by healthier women was a major barrier to implementation of Option B+. The WHO guidelines highlighted adherence and retention on antiretroviral therapy during the mother to child transmission risk period, and long-term retention of the women on lifelong antiretroviral therapy [4] as key operational challenges that needed to be addressed in the implementation of Option B+.

A study in Malawi found a higher rate of loss to follow up (LTFU) associated with a younger age at antiretroviral therapy initiation. This study also found significant proportions of women initiated antiretroviral therapy through Option B+ discontinued it (main reasons for discontinuation included travel away from home, lack of money for transport, development of adverse side effects, being too sick to travel to the clinic and limited understanding of the initial antiretroviral therapy education session) [8]. In Zimbabwe, a study on women receiving Option A prevention of mother to child transmission services by Muchedzi et al., found that living with a male partner, long waiting times at service delivery points, unreliable access to laboratory testing, high transport costs, perceived long queues at the health facility, competing life priorities (e.g., seeking food or shelter and inadequate referral information) were barriers to uptake and retention in prevention of mother-to-child transmission of HIV [9].

Understanding the acceptability of Option B+ to pregnant women was critical to strengthen the roll-out of the new guidelines, and inform health education and promotion activities as well as client counseling strategies for the new guidelines. We therefore undertook a qualitative study exploring the acceptability of Option B+ among pregnant and lactating women in Zimbabwe to inform health education and promotion activities and client counseling for the new guidelines in the country.

## Methods

### Study design

This qualitative study used in-depth interviews to give individual perspectives and focus group discussions to give insight into community perspectives on the acceptability of Option B+ among HIV positive pregnant and breastfeeding women in selected health facilities in Zimbabwe.

### Site selection

This study was conducted in health facilities in an urban district, which included the capital city of Harare, and in facilities in a rural district in the north west of the country. One facility still implementing Option A was included in this review, as well as six facilities that had transitioned to Option B+. Health facilities still implementing Option A were included to gather opinions from women who had not yet initiated Option B+ and to learn from them what they had heard about Option B+ in the community. Eight health facilities, four in each district, in the urban and rural districts were purposively selected for the study. All health facilities had the highest annual volume of HIV-positive pregnant women in these regions and had either to be still implementing Option A or had transitioned to Option B+.

### Study population

The study population included HIV-positive pregnant or breastfeeding women 18 years and older, attending antenatal care or postnatal care at the selected health facilities, on Option A or Option B+ and residing in the catchment area of the health facility for more than 6 months. Breastfeeding women who delivered a baby within 18 months prior to data collection (baby was currently alive, and breastfed) and had used Option B+ for a minimum of 3 months were also included. Healthcare workers (HCWs) from the study sites providing Option B+ participated as key informants. In health facilities implementing Option B+, participating health workers should have worked in the facility for the previous 6 months and initiated women on Option B+ for at least 4 months.

### Sample size

The number of in-depth interviews per site was 3–5 and the number of focus group discussions per site was 1–3. Each focus group discussion had a minimum of 5 participants to a maximum of 12.

### Participant eligibility criteria and recruitment process

Health workers identified HIV-positive pregnant and breastfeeding women eligible for the study in antenatal care and postnatal care and referred them to the research assistants. The research assistants used the client eligibility screening checklist to verify eligibility. If the woman was eligible, she was then asked to participate in the in-depth interview that was held the same day. If they said that they did not have the time to talk on that day, they were invited to participate in the focus group discussion, and informed of the later date at which it was held. When someone refused to be interviewed, the reason for refusal was recorded.

There were often only one to two nurses at each health facility; therefore for focus group discussion participation, additional nurses were recruited in the study from the maternal neonatal and child health department to get adequate numbers of health workers.

### Data collection methods

Data were collected by research assistants hired and trained by Elizabeth Glaser Pediatric AIDS Foundation from July 2014 to March 2015 (Table 1). Research Assistants were trained in human subject ethics and data collection methods. Each in-depth interview was conducted by a research assistant in a quiet place. Focus group discussions were conducted by two research assistants; one moderating and other taking notes and included 6–12 participants. Focus group discussions with health workers were conducted both in English and Shona. All in-depth interviews and focus group discussions with the women were conducted in Shona. In-depth interviews and focus group discussions were audio-recorded.

**Table 1:** Number of health facilities participating and in-depth interviews and focus group discussions conducted in the acceptability of Option B+ study implemented in Harare and Zvimba Districts, Zimbabwe, by study participant

Study Population	No. of health facilities	Total IDIs	Total FGDs
HIV-positive women who had not initiated Option B+	1	9	1
HIV-positive pregnant women who initiated Option B+	7	15	8
Breastfeeding women who have initiated Option B+	7	19	9
Health workers	5	0	4
Total	8	43	22

Topics explored in both in-depth interviews and focus group discussions included knowledge of HIV/AIDS, disclosure, acceptability of, perceived risks and benefits of lifelong antiretroviral therapy, social perceptions within the community on HIV and antiretroviral therapy, and respondent perceptions on the health facility’s ability to provide needed health services. Data collected from health worker focus group discussions explored challenges faced during transition to Option B+ implementation, challenges faced in delivering Option B+ services to patients, and barriers and facilitators for the women’s adherence to antiretroviral therapy under Option B+.

### Data analysis

All in-depth interviews and focus group discussion audio-recordings were translated from Shona to English (if needed) and transcribed by the research assistant who conducted the interview or discussion. Ten percent of the transcripts were checked to ensure accuracy of transcription and translation by the research coordinator. In-depth interview and focus group discussion transcripts were reviewed individually by two study team members to identify reoccurring themes and a code list was generated. Transcripts were uploaded into the software program MaxQDA version 10 (VERBI GmbH) and coded by two team members. During data analysis, the code list was modified twice to clarify code definitions. Data reduction and summary tables were generated to help identify recurrent themes. Using thematic analysis, the study team identified recurrent themes and selected representative quotes to illustrate the themes.

### Ethical considerations

All study research assistants were trained in the protection of human subjects in research and consenting process prior to contact with any study participants. Research assistants informed eligible participants about the purpose of the study, what it involved, confidentiality, participant’s rights and possible risks. Written informed consent was obtained from all study participants prior to data collection. The study was approved by the Institutional Review Board in Zimbabwe, the Medical Research Council of Zimbabwe.

## Results

### Women are accepting of Option B+

#### Women on Option A said Option B+ was not “too different” from Option A

The study participants from the 1 facility operating under an Option A policy felt that the Option B+ regimen was not very different from the Option A regimen and almost all women on Option A that were interviewed said they would accept Option B+. The women were motivated to accept Option B+ by the desire to stay healthy and have an HIV-negative baby.

*“No, it isn't that the medication is just similar to the ARVs, aren't they the same? So it’s ok to accept those (ARVs) as it is. Everything is easy. There is nothing difficult.”(Option A Woman in IDI).*

Seeing healthy women on Option B+ encouraged enrolment on Option B+. All women reported seeing the benefits of Option B+ and being motivated and willing to accept it when it is offered to them. Women were encouraged to initiate Option B+ when they saw other women who had initiated Option B+ looking healthy. This encouraged them to accept antiretroviral therapy in the hope they would experience similar benefits. While there were mixed views from the different study participants, most participants were accepting of Option B+. One woman on Option A said:

*“Eh! When you know someone’s on HIV medicine, you compare the kind of life that he/she is leading and yours and sometimes you notice that the person on HIV medicine is healthy. So, this helps you to find it easy to accept the offer to be initiated on HIV medicine. You will say even so and so is on HIV medicine but they are leading a good life.” (Option A Woman in IDI).*

Knowing women who are HIV-positive, have accepted their status, and are adherent on ART also encouraged some women to initiate and adhere to lifelong antiretroviral therapy. One lactating woman said:

*“Yes, it encourages, because if you hear from their experiences, you learn from what they would have gone through, how it helped them, where they came from and where they are going.” (Lactating Woman in FGD).*

### Health care Worker’s concerns about Option B+

While HCWs felt that women were generally accepting of Option B+, they discussed challenges of women receiving insufficient counseling due to insufficient staff.

*…there are some who just come, who are not yet ready to accept ART, you find that those people need a lot of counselling so, in short I can say for the healthcare worker, it’s quite difficult because the workload is too much. (HCW in FGD).*

HCWs primary concern in provision of Option B+ was the lack of HCWs to provide sufficient counseling to ensure the women understand Option B+. Lack of HCW manpower was a serious concern expressed by many HCWs.

*The issue of service provision is a bit of a challenge because, we have few nurses and the option B+ program is an addition to our work yet the number of nurses did not increase, so like what has been said by other participants there is need for more time with these people, but due to our programs we fail to even squeeze the time for special cases [women struggling to accept ART]. (HCW in FGD).*

### Option B+ reduces stigma experienced by HIV-positive women

Both women and health workers from all seven health facilities felt that Option B+ reduced stigma towards HIV-positive people. Many participants felt that the physical symptoms of being HIV-positive were much less visible now.

*“We no longer get wasted, and nowadays no one can tell the difference between an HIV-positive and an HIV-negative person. We all look the same…” (Lactating woman in FGD).*

Another benefit of Option B+, welcomed by the women, was the ability to continue breastfeeding after 6 months. The fact that HIV-positive women could breastfeed for as long as HIV negative women also seemed to reduce stigma associated with community-suspected HIV infection.

*“It is good, because those who were breastfed during that time when the baby was supposed to stop breastfeeding after 6 months all the people would know, even those who were not aware would just know that this one who has stopped breastfeeding at 6 months is HIV-positive, but currently we are all the same, we are taking the medicine for the rest of our lives and I will breastfeed my child until I feel that it’s enough. Therefore this medicine is good.” (Lactating Woman in FGD).*

### Option B+ allows women to lead normal “healthy” lives

One of the most commonly cited benefits of Option B+ was women reporting strengthened health. Many lactating women felt very healthy on Option B+, which then allowed them to take care of their families.

*“I wanted to say that we accept it well because lifelong antiretroviral therapy keeps us healthy all the time and we are able to do our daily chores without being sick all the time since we will be taking our medication well.” (Lactating Woman in FGD).*

Health workers commented that women preferred Option B+ because they would rather take the drugs themselves than to give the infant drugs.

*“Several women I spoke to felt that it was better for them to take the medication than to have babies take the medication. They said if it is difficult for adults, how much more will it be for the infants. It is also us adults that pass on the virus to the infants.” (Health Worker in FGD).*

### To strengthen Option B+, we need to strengthen community sensitization

Despite the acceptance of Option B+ among pregnant, lactating women and health workers, there remained a need to gain acceptance among the general community and men.

*“Mostly if they hear that you are on HIV medicine they start talking badly about you behind your back, but they don’t know that there are many benefits from using this medication.” (Pregnant Woman in FGD).*

In every program or activity that involves communities, community sensitization is an important aspect of success. Sensitization helps people understand the new activity and dispels myths and misconceptions. Both pregnant and lactating women expressed that some people who are uninformed about ART speak poorly about antiretroviral therapy and looked down on those using it. Study participants advocated for stronger sensitization of community members, specifically through education campaigns.

*“In my view, there is need for ongoing education campaigns in the community so that they [community members] do not fear or find it hard to come to the hospital to initiate lifelong antiretroviral therapy.” (Pregnant Woman in FGD).*

*“I think the campaign part of it is very important, whereby we use posters, t-shirts with campaign messages. Umm, like we have seen this happen with male circumcision program. If things are campaigned for, people tend to opt in but if it happens within smaller groups, it is difficult for this information to reach people. So campaign in any form, e.g. t-shirts, posters, caps and so forth can be helpful to spread these messages and encourage men to accompany their wives for antenatal care.” (Health Worker in FGD).*

### Strengthening male engagement is critical under Option B+

Health workers and women both discussed the need for educating men about HIV and antiretroviral therapy. Health workers reported low male involvement in their clinic settings. Health workers commented that the current methods of community outreach may not be successful and more outreach by health workers to communities may be necessary.

*“I noticed that the people who are getting information are women mostly because when they go to the clinic especially when pregnant, they get taught on these things. Men are not getting this information. It would be good to have certain groups that go to the beerhalls to join the men and teach them on acceptance.” (Lactating Woman in FGD).*

As a result of men lacking knowledge about HIV and antiretroviral therapy, women struggle to disclose their HIV status to their partners.

*“The other challenge is that of disclosure whereby the mother is tested but she cannot disclose to her husband, so we have been encouraging mothers to come with their husbands so that we can disclose the results to them as a couple. If they come as a couple and one of them tests HIV-positive it becomes easy for them because they will remind each that to take the medicine and the one taking the medicine will feel free to take the medicine since the partner will be aware that my partner is taking medicine.” (Health worker in FGD).*

With the arrival of Option B+, disclosure has become a more important issue to address, because it is challenging for women to hide long term use of antiretroviral therapy. Men have influence over many decisions in women’s lives. When men do not understand ART they may try to force their female partner to make incorrect decisions. Men may tell their spouse to stop taking HIV medicine, if they think they look healthy.

*“Very few men are supportive. You have to be strong. The men base their judgment on how healthy you appear to be as you carry yourself around and he also compares to how healthy he feels and opts to delay testing. But delaying only brings further harm. So when those men tell you to stop taking your medication, you need to tell them that they can stop if they want to, whilst you continue with your treatment.” (Lactating Woman in FGD).*

## Discussion

One of the most important findings in this study is the general acceptance of lifelong antiretroviral therapy under Option B+. All women generally felt Option B+ was not “too different” from Option A and it was not going to be a challenge to transition from Option A to B+. Women were encouraged to initiate antiretroviral therapy by seeing others who were healthy on treatment, and by seeing other HIV-positive women on ART having HIV-negative babies. Option B+ allowed women to regain their health and lead “normal” lives. Most women cited strengthened health as a benefit of accepting Option B+ allowing them to take care of their families and perform all others chores expected of them just like healthy HIV-negative women. Most also mentioned the reduction in stigma, particularly around continued breastfeeding as a benefit of Option B+.

Previously, limited research had shown that the healthy pregnant and lactating women may be resistant to initiating life-long ART, with stigma being one of the most common reasons to refuse antiretroviral therapy. Several studies have shown that women have refused to initiate Option B+ because they felt healthy [10–12]. In a study by Katz et al., in Soweto, the major reason for rejection of antiretroviral therapy initiation was “feeling healthy” even when their CD4 cell counts were low and after 2 months of counseling [13].

Stigma associated with HIV and AIDS is a key social factor in limiting acceptance of and successful completion of PMTCT interventions. In a study by Ilwelunmor et al., HIV was synonymous to death among people living with HIV [14]. HIV has been labeled as a disease of the immoral [15]. In Kabarole, Uganda, HIV positive people were viewed as “walking corpses” [16] while in Mali, people believed those with HIV were receiving punishment from God [15, 17–20].

Our study highlights a shift in the perception of HIV and the use of antiretroviral therapy, with more women being willing to initiate ART with the perception that treatment is a “good” option that will allow them to lead a healthy life. One of the reasons for the decrease in stigma and general acceptance of ART is the perception that antiretroviral therapy now makes one look “normal” and “healthy” as opposed to having negative effects on physical appearance.

HIV-related stigma tends to put breastfeeding women at risk of non-adherence to antiretroviral therapy [21]. Another aspect which helped reduce stigma under Option B+ was the new breastfeeding guidance that allowed an unrestricted breastfeeding period and a much shorter period of administering nevirapine to the infant. Under Option A, many women stopped breastfeeding at 12 months or earlier, which raised suspicion and concern among family and community members when women appeared to stop breastfeeding earlier than the general population, where breastfeeding often continued through 24 months. Option B and B+ allowed breastfeeding duration to be similar to the general population and infants only receive nevirapine for 6 weeks following birth, as the infants would be protected by continued maternal antiretroviral therapy. The women and health workers interviewed indicated a strong preference to women administering the drugs to themselves rather that to their infants for extended periods of time [7].

While HIV-positive women who participated in the study generally accepted Option B+, it was apparent from this study that the general knowledge and acceptance of antiretroviral therapy in the community needs strengthening. Women who access the facility and counseling services generally have positive attitudes towards antiretroviral therapy but there is a large knowledge gap in the community which may deter others from accessing the facility and may make it more challenging for women to disclose to their partners. A study in Vietnam that provided community leaders with HIV knowledge and sensitized them on impact of HIV stigma followed by implementing a package of community sensitization activities resulted in a decline in stigma scores compared to the period before the intervention [22]. Additionally, communities in Nigeria sensitized through mass media communication were associated with significant and positive trend in acceptance of people living with HIV (3.5% to 9.0% Pearson’s χ^2^ trend *p* < 0.0001) [23]. Gliemann and Muchedzi et al. in Zimbabwe showed that incorporating a community sensitization component was effective in creating demand for antenatal care and PMTCT services among pregnant and postnatal women and increased women’s awareness of HIV transmission risk [24, 25]. Community sensitization can reduce HIV stigma in the community and thereby make disclosure more possible for women. To strengthen Option B+ in Zimbabwe, community sensitization needs to receive higher priority and more resources.

Previous research has shown that male support strengthens women’s adherence to prevention of mother to child transmission of HIV [26]. In our study, health workers reported low male involvement in PMTCT services, which most women felt was due to men’s lack of knowledge. In addition, men and the Zimbabwean society in general perceive antenatal care and prevention of mother to child transmission of HIV as a woman’s activity, and it was unacceptable for men to be involved. Women, because of their health seeking behavior, especially around pregnancy, tend to receive HIV education at the facility while men who view illness as a “women’s issue” remain uneducated about HIV. This may cause problems when men fail to understand their spouses and support them in care.

There have been conflicting results in the literature around the significance of male involvement in prevention of mother to child transmission of HIV. In one study, community sensitization did not increase male partner involvement at antenatal care and HIV testing uptake [27]. However, another study in Zambia found engaging and sensitizing the community by health workers focusing on male partner involvement was associated with twofold increase in women accepting HIV testing and counseling and ARV prophylaxis [28]. Farquhar et al. noted that the sensitization and engagement of male partners resulted in a significant increase in the acceptance of ARV prophylaxis among pregnant women and partner participation in counseling and testing sessions [29].

In various studies, disclosure of HIV status to the spouse or a close relation enabled women to adhere to antiretroviral therapy during their pregnancy [30, 31]. Our study is consistent with this finding. In practice, the male partners tend to have the final decision in most matters between them and their spouses. Women may therefore need to consult husbands to get their permission to enroll and initiate on antiretroviral therapy and get their support in adherence. Men also usually have control over the financial resources that women need to reach the health facilities. With the lifelong commitment of antiretroviral therapy, sensitizing men and gaining an overall understanding of HIV and antiretroviral therapy will be critical.

### Limitations

The perspectives of the study population are limited to HIV-positive pregnant women accessing antenatal care and therefore do not express the opinions and perspectives of women who were not attending health facilities. The perspectives of those accessing the facility are also limited to those who accepted antiretroviral therapy (or ARV prophylaxis under Option A). Additionally, no data were collected from male partners; we only received the perspectives of female partners and health workers on perceptions of male engagement in maternal health issues.

## Conclusion

This study has found that Option B+ is generally accepted among HIV-positive pregnant women as a means to strengthen their own health and protect their babies from HIV infection. Consistent with previous literature, the study demonstrated the importance of male partners supporting their partners for satisfactory adherence to antiretroviral therapy. Strengthening community sensitization and male knowledge is critical to encourage women to disclose their HIV status and ensure successful adherence to antiretroviral therapy. Targeting and engaging the partners of the women will remain a key determinant to women’s acceptance and adherence on antiretroviral therapy under Option B+.

## Abbreviations

AIDS: Acquired immune deficiency syndrome
ANC: Antenatal care
ART: Antiretroviral therapy
ARV: Antiretroviral drugs
AZT: Zidovudine
FGD: Focus group discussion
HIV: Human immune virus
IDI: In-depth interview
LTFU: Loss to follow up
MTCT: Mother-to-child transmission
NVP: Nevirapine
PMTCT: Prevention of mother-to-child transmission
PNC: Postnatal care
RA: Research assistant
WHO: World Health Organization
